# Effect of tai chi on musculoskeletal health-related fitness and self-reported physical health changes in low income, multiple ethnicity mid to older adults

**DOI:** 10.1186/1471-2318-13-114

**Published:** 2013-10-28

**Authors:** James Manson, Michael Rotondi, Veronica Jamnik, Chris Ardern, Hala Tamim

**Affiliations:** 1 York University, 341 Bethune College, 4700 Keele Street, M3J 1P3 North York, Ontario, Canada; 2 University of Western Ontario York University, 341 Bethune College, 4700 Keele Street, M3J 1P3 North York, Ontario, Canada; 3 University of Toronto, York University, 341 Bethune College, 4700 Keele Street, M3J 1P3 North York, Ontario, Canada; 4 Queen’s University, York University, 341 Bethune College, 4700 Keele Street, M3J 1P North York, Ontario, Canada; 5 McGill University, York University, 341 Bethune College, 4700 Keele Street, M3J 1P3 North York, Ontario, Canada

**Keywords:** Ageing, Community dwelling, Ethnicity of origin, Health-related fitness, Low income, Tai Chi

## Abstract

**Background:**

Tai Chi (TC) has proven to be effective at improving musculoskeletal fitness by increasing upper and lower body strength, low back flexibility and overall physical health. The objectives of this study were to examine changes in musculoskeletal health-related fitness and self-reported physical health after a 16 week TC program in a low income multiple ethnicity mid to older adult population.

**Methods:**

Two hundred and nine ethnically diverse mid to older community dwelling Canadian adults residing in low income neighbourhoods were enrolled in a 16 week Yang style TC program. Body Mass Index and select musculoskeletal fitness measures including upper and lower body strength, low back flexibility and self-reported physical health measured by SF 36 were collected pre and post the TC program. Determinants of health such as age, sex, marital status, education, income, ethnicity of origin, multi-morbidity conditions, weekly physical activity, previous TC experience as well as program adherence were examined as possible musculoskeletal health-related fitness change predictors.

**Results:**

Using paired sample t-tests significant improvements were found in both upper and lower body strength, low back flexibility, and the SF 36 physical health scores (p < 0.05). Based on multiple linear regression analyses, no common health determinants explained a significant portion of the variation in percent changes of the musculoskeletal fitness and SF 36 measures.

**Conclusions:**

These results reveal that TC has the potential of having a beneficial influence on musculoskeletal health-related fitness and self-reported physical health in a mid to older low socioeconomic, ethnically diverse sample.

## Background

As the Canadian population both ages and increases in number musculoskeletal health-related fitness maintenance concerns become a higher priority. Physical activity (PA) has been shown to be effective in the prevention and management of cardiovascular disease, stroke, hypertension, breast cancer, colon cancer, type 2 diabetes and osteoporosis [1]. Since many older adults must deal with multiple chronic health conditions that may place them close to important thresholds of physical ability that straddles the line between independence and dependence [2] it is important to research and implement appropriate community based activity programs. Adding further layers to this challenge are health-related fitness concerns that are unique to specific populations. Data has consistently shown low socioeconomic status (SES), ethnic minorities and new immigrants have lower activity levels than White or non-immigrant groups [3] as well as living in poorer social environments that add to the barriers of activity overall [4].

Tai Chi (TC) has been shown to be a successful program for reduction in falls, health-related cardiovascular and musculoskeletal fitness, arthritis and psychosocial behavior [5]. In TC, the body is comfortably moved and relaxed, the mind is focused on the moment, and body movements are slow, smooth, and well-coordinated as the various forms are undertaken [6]. TC exercise has the ability to produce balanced movements between natural physical and metabolic processes in the body; in a slow, meditative, and relaxed way. These sequential graceful movements emphasize the smooth integration of trunk rotation, weight shifting, and coordination, along with a progressive narrowing of one’s stance or base of support. The intensity of TC is moderate and approximately equivalent to a walking speed of 3.7 mph [7]. As powerful as TC is as a good, low-intensity exercise, it is important to emphasize the social benefits as part of the participation structure that helps keep the mind engaged, combined with this, evidence has demonstrated that being active with people of similar age, ability and outlook highly influences the social rewards that are a significant factor for adherence to long-term practice [8]. Physically TC is highly appropriate for an aging population since it can be practiced by participants with one or more chronic conditions due to its low intensity, steady rhythm and low physical and mental demands but it can also specifically influence balance and motor control to reduce falls in this at risk aging population group [9].

Although several studies to date have reported musculoskeletal health-related fitness benefits for mid to older adults who practice TC (community living and institutionalized) [10], they have not specifically focused on low socioeconomic status (SES) and ethnically diverse aging populations who could also benefit substantially from an inexpensive, low impact PA program such as TC. Factors such as age, sex, marital status, among others, may individually or collectively influence these health-related musculoskeletal fitness outcomes [11]. The above notwithstanding, the goal with any PA modality is to improve overall health-related fitness. In this sense, the maintenance of adequate musculoskeletal fitness allows older adults to perform normal daily activities in a safe and independent fashion without undue fatigue or pain [12].

The primary objective of the current study was to examine the effect of a 16 week TC program on musculoskeletal health-related fitness and self-reported physical health changes in a sample of low SES, ethnically diverse mid to older community dwelling Canadian adults. As a secondary objective we aimed to identify factors related to overall changes in musculoskeletal health-related fitness and SF 36 physical health improvement.

## Methods

The study targeted community dwelling mid to older adults of various ethnicities of origin in two locations in the Greater Toronto Area of Ontario, Canada; Jane and Finch as well as Dundas and Spadina. These two locations were chosen for their diversity of ethnic groups represented and their low SES. The city of Toronto has specific areas that have a significant proportion of socially and economically vulnerable population groups, the two areas chosen for this study were almost identical in low income rates [13]. The Dundas and Spadina and Jane and Finch area have a population average income of about $26,771.00 [14]. Ethnic or visible minorities in Canada are defined as persons, other than Aboriginal peoples, who are non-Caucasian in race or non-white in color [15]. The Jane-Finch community is ethnically diverse and has approximately 86,000 immigrants with 63% of the total population is a visible minority [16]. Poverty and various forms of discrimination, including racism, have been identified as negatively affecting the quality of life of the community’s families, and as significant risk factors for poor physical and mental health [17]. Moreover, the SES of the Jane-Finch population is modest compared to many other areas of Toronto and Ontario as a whole [13]. Dundas and Spadina was a logical choice since it is an area that is particularly dense in adults of Chinese origin (a purposeful decision to explore research questions of ethnic origin affiliations with TC) as well as being socio-economically similar to the Jane-Finch community [13]. Almost 50% of Chinese Canadians live in Ontario, and the Dundas-Spadina area of Toronto, is identified as the center of one of the largest Chinese communities in North America [18].

Eligibility for participation was limited to those individuals who were 50 years of age and older (male/female), residing in both target community, and who had the medical capability to be involved in an physical activity program. This capability was measured by Physical Activity Readiness Questionnaire (PAR-Q) which is a self-screening tool that if any participant answers yes to one of the seven health questions they must be cleared by their doctor via the Physical Activity Readiness Medical Examination (PAR-Med-X)[19].

To facilitate enrollment and to increase access to the TC programs, two focus groups (male/female) were conducted for each cohort to identify barriers and promoters to participation in a community based TC program. The focus group attendees were recruited from the local community and key contacts from community housing agencies in the geographical area. Information attained from participants of these focus groups helped identify information dissemination strategies (and areas) in the Jane-Finch and Dundas-Spadina neighborhood for targeted recruitment. In addition to these strategies, further recruitment of study participants was achieved through networking and invitations from focus group participants. Three cohorts of participants were followed; cohorts 1 and 3 were recruited from the Jane and Finch area and were followed from August through December 2009, and from October 2011 through April 2012 respectively. Participants for cohort 2 were recruited from the Dundas and Spadina area and were followed from February through August 2011. For cohort 1, participant recruitment was completed prior to commencement of the TC program whereas for cohorts 2 and 3, participants were enrolled on an ongoing basis, which accounts for the 6-month duration. All participants were exposed to 16 consecutive weeks of TC.

### Program

For each of the three cohorts, a TC program was offered free of charge to the participants. The TC program consisted of 6-7 classes given throughout the week where participants were advised to attend two classes per week for 16 consecutive weeks. Classes for cohort 1 took place at a Toronto Community Housing building whereas classes for cohorts 2 and 3 took place at local community centers in their respective area. A professional Tai Chi master facilitated the classes. Each class was 60 minutes long and consisted of 15 minutes of a Qigong warm up followed by 45 minutes of Yang style TC. A research assistant monitored participation in the TC classes so that exact attendance could be recorded.

### Study variables

Socio-demographics and physical health-related fitness characteristics were collected at baseline and included information on sex, age, education, smoking/drinking status, marital status, income and chronic conditions. Weekly PA, based on the CPAFLA Healthy Physical Activity Participation that examines frequency, intensity and perceived fitness, - and previous lifetime TC participation of more than one year, were also recorded [19]. Pre- and post-TC program musculoskeletal health-related fitness characteristic testing was conducted by qualified exercise personnel and were assessed pre- and post-TC program by employing a combination of the Canadian Physical Activity Fitness and Lifestyle Approach and the Senior Fitness Test [12,19]. These measures included anthropometrics (height and weight which was used to calculate body mass index), upper body (overall grip strength, arm curl test in 30 seconds), lower body (chair stand test in 30 seconds, timed 8-foot up and go test) and lower back flexibility measure (sit-and-reach). Height was measured using a wall mounted tape measure without footwear, standing erect, arms hanging by the sides with feet together, the heels and back in contact with the wall using a set square the measure was made to the nearest 0.5 cm. Weight was measured using a calibrated scale on a wooden surface with the participant wearing light clothing, the weight in kilograms was measure to the nearest 0.1 kg. Grip strength was taken using a dynamometer using each individual hand allowing for two trials with the maximum of each combined together. The arm curl test in 30 seconds was done with the participant sitting on a chair with back straight and feel flat on the floor and the dominant side of the body close to the edge of the seat. For men the weight was 3.63 kg pounds and for women the weight was 2.27 kg and was held in the dominant hand, perpendicular to the floor with a handshake grip. Participants were allowed two repetitions without the weight to ensure proper form before the test of as many curls as possible in 30 seconds. The chair stand test in 30 seconds involved the participant siting in the middle of the chair with back straight, feet flat on the floor, arms crossed at the wrist and held against the chest. On the signal “go” the participant rose to a full stand, then returned to a fully seated position, the participant was allowed two stands to ensure proper form. The timed 8-foot up and go test had the participant sitting in a chair that was placed against a wall and facing a cone marker exactly 8 feet away, on the signal “go” the participant got up from the chair, walked as quickly as possible around either side of the cone and sat back down in the chair. The participant was allowed one test practice and then two test trials. The sit and reach test involved a small warm up of a modified hurdle stretch held for 20 seconds for each leg before they placed their feet, without shoes, against the flexometer. The participants was coached to reach forward along the flexometer ruler while breathing out and extending their arms, palms down to a comfortable limit that was held for two seconds. This test was repeated twice. The SF-36 scales of physical functioning, role-physical, bodily pain and general health were assessed pre- and post-TC program were used to provide an overall summary measure of self-reported physical health [20].

### Statistical analysis

Differences in baseline socio-demographic, physical health related characteristics, musculoskeletal health and SF-36 physical scales among participants by cohort was performed using chi square tests and a one-way ANOVA. To compare the health-related musculoskeletal fitness measures and SF 36 physical scales for the pre versus post TC program values pair samples t-tests were performed for both the individual cohorts and the combined cohorts, effect size was determined using a Cohen’s D calculation. To examine the determinants of health predictors of changes for these outcomes, a multivariate linear regression model was performed for each of the health-related musculoskeletal fitness dependent variables and the overall summary measure of the SF 36 scales. For each of the regression models the dependent variable was the percent change (calculated as the post minus the pre score divided by the pre score and multiplied by 100) and the independent variables included age, sex, marital status, education, income, ethnicity of origin (defined by Chinese versus non-Chinese origin), attendance, previous TC experience, weekly physical activity and multi-morbidity influences. Standardized beta coefficients and R^2^ were reported. Significance was set at an alpha of 0.05. The study was approved by the human participants ethics review committee of York University.

## Results

**Figure 1:**
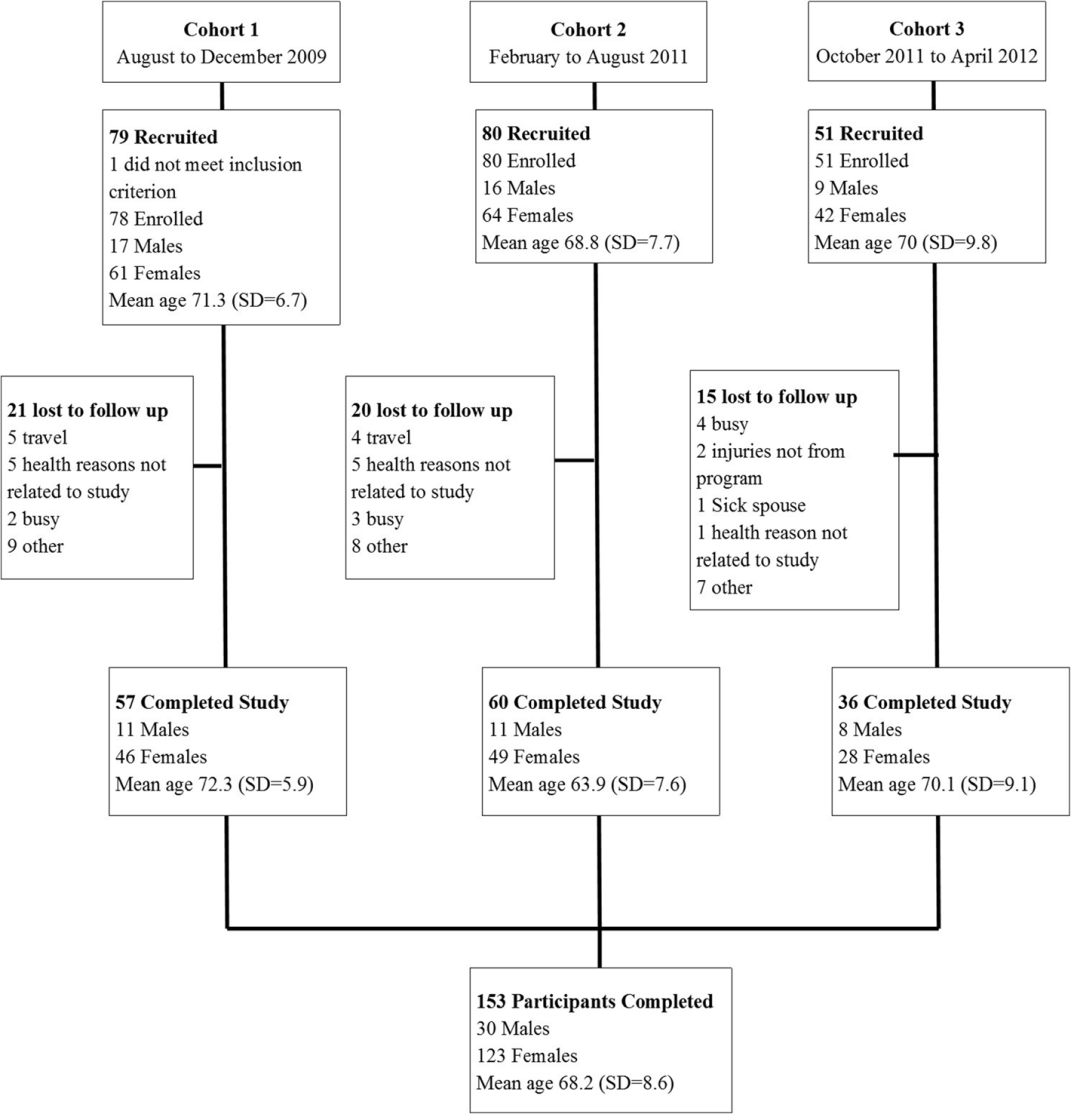
Flowchart of study.

A total of 209 participants were recruited for this study (78, 80, and 51 for cohorts 1, 2, and 3 respectively). Figure 1 shows the study flow, recruitment, enrollment and loss to follow up. Of the 209 overall sample recruited, 56 (26.7%) did not complete the study and were lost to follow-up. Reasons for loss to follow-up included health reasons not related to the TC program, travel, busy or inaccessible for post TC program data collection and unknown reasons.

Tables 1 and 2 summarize the socio-demographic characteristics, physical characteristics, health-related musculoskeletal fitness characteristics and SF 36 physical scales of the 209 study participants. There were initially 79.9% female and 20.1% male participants and the overall mean age of the participants at enrollment was 68.1 years (range 50-87 years). The ethnicity of origin of participants included China (36.1%), South America (26.3%), Caribbean (6.3%), Europe (16.1%), South Asia (4.9%), Canada (6.2%) and other (3.9%).

**Table 1:** **Baseline socio**-**demographic & physical characteristics of program participants**

	**Combined cohorts N (%)**	**Cohort 1 N (%)**	**Cohort 2 N (%)**	**Cohort 3 N (%)**	**P**
**Totals**	**209 (100.0)**	**78 (37.3)**	**80 (38.2)**	**51 (24.4)**	
**Sex**					
Male	42 (20.1)	17 (21.8)	16 (20.0)	9 (17.6)	
Female	167 (79.9)	61 (78.2)	64 (80.0)	42 (82.4)	.847
**Age groups**					
50-64 years	73 (35.3)	12 (15.4)	46 (57.5)	15 (30.6)	
65-74 years	86 (41.5)	41 (52.6)	28 (35.0)	17 (34.7)	<0.001
75+ years	48 (23.2)	25 (32.1)	6 (7.5)	17 (34.7)	
*Mean* (*SD*)	*68*.*1* (*8*.*62*)	*71*.*3* (*6*.*7*)	*63*.*8* (*7*.*7*)	*70* (*9*.*8*)	<0.001
**Ethnicity of origin**					
Chinese	74 (36.1)	0 (0)	73 (91.3)	1 (2.0)	
South American	54 (26.3)	45 (60.8)	2 (2.5)	7 (13.7)	
Caribbean	13 (6.3)	9 (12.2)	1 (1.3)	3 (5.0)	<0.001
European	33 (16.1)	7 (9.5)	2 (2.5)	24 (47.1)	
South Asian	10 (4.9)	10 (13.5)	0 (0)	0 (0)	
Canadian	13 (6.3)	1 (1.4)	0 (0)	12 (23.5)	
Other	8 (3.9)	2 (2.6)	2 (2.5)	4 (7.8)	
**Education**					
< High School	94 (46.5)	47 (61.0)	33 (41.2)	14 (28.6)	
High School	79 (39.1)	25 (32.5)	34 (44.7)	20 (40.8)	<0.001
> High School	29 (14.3)	5 (6.5)	9 (11.8)	15 (30.4)	
Smoking Status-Yes	4 (1.9)	2 (2.6)	1 (1.3)	1 (1.9)	.833
Drinking Status-Yes	45 (21.4)	17 (21.8)	9 (11.3)	19 (36.5)	.003
**Marital status**					
Single	112 (54.9)	59 (75.6)	27 (35.1)	26 (52.0)	<0.001
Married	90 (44.1)	18 (23.1)	50 (64.9)	23 (48.0)	
**Income**					
<$14,000 per year	135 (71.4)	66 (90.4)	52 (70.3)	17 (40.5)	
$14,000-$30,000	35 (18.5)	5 (6.8)	12 (16.2)	18 (42.9)	<0.001
>$30,000	19 (10.1)	2 (2.7)	10 (13.5)	7 (16.7)	
**Chronic conditions**					
Hypertension	105 (50.0)	52 (49.4)	27 (33.8)	26 (50.0)	<0.001
Arthritis	102 (48.6)	38 (51.3)	39 (48.8)	25 (48.1)	.997
Diabetes Mellitus	45 (21.4)	28 (19.5)	9 (11.3)	8.(15.4)	<0.001
Sleep Disturbance	54 (25.7)	20 (25.6)	21 (26.3)	13 (25.0)	.987
Depression	31 (14.8)	14 (17.9)	5 (6.3)	12 (23.1)	.017
Hearing Impairment	26 (12.4)	9 (11.5)	9 (11.3)	8 (15.4)	.749
Disorientation	14 (7.2)	9 (11.5)	4 (5.0)	1 (1.9)	.090
Heart Disease	12 (5.7)	8 (10.3)	3 (3.8)	1 (1.9)	.097
^a^COPD	10 (4.8)	4 (5.1)	2 (2.5)	4 (7.7)	.385
Tumour	5 (2.4)	1 (1.3)	0 (0.0)	4 (7.7)	.013
^b^Multi-Morbidity	133 (63.3)	56 (71.8)	41 (51.2)	36 (69.2)	.016
Walking Assistance	18 (8.6)	13 (16.7)	0 (0.0)	5 (9.6)	.001
^**c**^**Weekly physical activity**					
Needs improvement/fair	35 (23.3)	14 (18.4)	15 (20.0)	6 (12.5)	
Good	16 (7.9)	8 (10.5)	4 (5.3)	4 (8.3)	.606
Very good/excellent	150 (74.6)	56 (71.0)	56 (74.7)	38 (79.6)	
*Mean* (*SD*)	*6*.*7* (*3*.*1*)	*6*.*4* (*2*.*9*)	*6*.*6* (*3*.*1*)	(79.6)	.*196*
^**d**^**Previous Tai Chi**	38 (18.1)	0 (0.0)	32 (40.0)	6 (11.8)	<0.001

^a^*COPD*: chronic obstructive lung disease.

^b^Multi-morbidity: two or more chronic conditions.

^c^Physical Activity: based on the Healthy Physical Activity Participation Questionnaire.

^d^Previous Tai Chi: Previous Tai Chi participation more than one year.

Note: Totals may vary due to missing responses.

**Table 2:** **Baseline health**-**related musculoskeletal fitness characteristics and SF 36 physical scales and physical summary measure of study participants**

	**Combined cohorts mean (SD)**	**Cohort 1 mean (SD)**	**Cohort 2 mean (SD)**	**Cohort 3 mean (SD)**	**P**
**Anthropometric measures**					
Body Mass Index (kg/m^2^)	26.7 (4.8)	28.3 (4.8)	24.3 (3.7)	28.2 (5.2)	<0.001
**Upper body musculoskeletal measures**					
Overall Grip Strength (kg)	54.3 (17.7)	46.8 (16.8)	59.8 (16.5)	57.7 (16.8)	<0.001
Arm Curl Test in 30 Seconds (#)	15.5 (5.4)	11.9 (4.0)	17.9 (4.4)	17.6 (5.9)	<0.001
**Lower body musculoskeletal measures**					
Chair Stand Test in 30 Seconds (#)	12.2 (4.1)	10.0 (3.1)	13.9 (4.1)	13.2 (3.8)	<0.001
Timed 8-Foot Up and Go (secs)	7.6 (3.2)	8.8 (4.2)	6.4 (1.7)	7.8 (2.2)	<0.001
**Low back flexibility measures**					
Sit and Reach (cm)	26.4 (9.1)	25.3 (8.2)	26.2 (9.6)	28.7 (9.1)	.141
**SF 36 physical scales**					
Physical Functioning (PF)	75.0 (21.6)	67.2 (23.8)	80.9 (16.4)	78.0 (22.1)	<0.001
Role-Physical (RP)	80.9 (25.6)	74.4 (29.5)	87.0 (21.1)	81.8 (23.1)	.009
Bodily Pain (BP)	68.7 (24.8)	62.8 (27.2)	74.1 (22.7)	69.4 (22.2)	.017
General Health (GH)	64.8 (20.5)	64.9 (21.1)	60.5 (20.2)	71.7 (18.3)	.012
**SF 36 physical summary measure**					
Physical Health (PCS)	49.2 (7.9)	46.9 (8.8)	50.7 (5.9)	50.3 (8.6)	.009

Note: # designates repetitions completed.

Several differences existed between cohorts notably with cohort 2 having a lower mean age of 63.8 years of age. Cohort 3 also had a higher prevalence of (greater than high school) education (30.4%), whereas cohort 2 had a higher percentage of married participants (64.9%). Finally, cohort 1 had the greatest proportion of lower income status (less than $14,000) participants (90.4%).

Baseline musculoskeletal fitness characteristics for the overall cohort can be seen in Table 2. Differences between musculoskeletal fitness characteristics between cohorts were also observed, cohort 1 having a lower upper body strength (overall grip strength 46.8 ± 16.8 kg and arm curl test in 30 seconds 11.9 ± 4.0). Cohort 1 also was below the other cohorts in lower body musculoskeletal strength (chair stand test in 30 seconds 10.0 ± 3.1 and timed 8-foot up and go 8.8 ± 4.2). As well in the SF 36 physical summary measure cohort 1 had a lower mean score of 46.9 ± 8.8.

For the overall sample, mean attendance was 1.1 ± 0.94 sessions per week with 0.9 ± 0.72, 0.9 ± 1.44, and 1.3 ± 0.86 for cohorts 1, 2 and 3 respectively.

Table 3 summarizes the differences in effect size using Cohen’s D for all outcomes. Participation in the 16-week TC program showed no significant change in body mass index measures, role physical and bodily pain. However, significant improvements were observed in musculoskeletal health measures of overall grip strength, arm curl in 30 seconds, chair stand in 30 seconds, 8-foot up and go test and sit and reach as well as physical functioning, general health, and the aggregate summary measure of physical health in the SF 36 (p < 0.05).

**Table 3:** **Mean difference of health**-**related musculoskeletal fitness measures**, **SF 36 physical scales and summary measure**

	**Combined cohorts Cohen’s D**	**P**	**Cohort 1 Cohen’s D**	**P**	**Cohort 2 Cohen’s D**	**P**	**Cohort 3 Cohen’s D**	**P**
**Anthropometric measures**								
Body Mass Index (kg/m^2^)	-0.048	.450	0.100	.906	-0.273	.004	-0.778	.002
**Upper body musculoskeletal measures**								
Overall Grip Strength (kg)	0.312	<0.001	0.420	.002	0.188	.262	0.298	.133
Arm Curl Test in 30 Seconds (#)	0.673	<0.001	0.895	<0.001	0.661	<0.001	0.404	.034
**Lower body musculoskeletal measures**								
Chair Stand Test in 30 Seconds (#)	0.717	<0.001	0.957	<0.001	0.790	<0.001	0.676	.001
Timed 8-Foot Up & Go Test (secs)	-0.300	.001	-0.214	.321	-0.533	<0.001	-0.231	.047
**Low back flexibility measures**								
Sit & Reach (cm)	0.268	.004	0.155	.315	0.188	.167	0.575	.009
**SF 36 physical scales**								
Physical Functioning (PF)	0.326	<0.001	0.355	.006	0.326	.004	0.276	.180
Role Physical (RP)	0.037	.663	-0.069	.696	0.004	.918	0.368	.046
Bodily Pain (BP)	0.047	.587	-0.087	.557	0.051	.655	0.300	.069
General Health (GH)	0.241	.005	0.252	<0.001	0.209	<0.001	0.284	<0.001
**SF 36 physical summary measure**								
Physical Health (PCS)	0.192	.033	0.067	.582	0.209	.025	0.487	.055

Cohen’s *D*: mean difference calculated as post measure minus pre measure divided by standard deviation.

Note: # designates repetitions completed.

Table 4 shows results of the multivariate linear regression models. Overall, no common health determinants explained a significant portion of the variation in percent changes of the musculoskeletal health-related fitness and SF 36 physical health measure. Percent change in body mass index, overall grip strength, arm curl in 30 seconds, chair stand in 30 seconds and the 8-foot up and go test all showed a significant linear association (p < 0.05) with a limited number of predictors. The sit and reach test and the physical health summary measure showed no significant linear associations’.

**Table 4:** **Multivariate linear regression for relationship between musculoskeletal health**, **SF 36 physical summary measure and determinates of health**

	**Body mass index (kg/m**^**2**^**)**		**Overall grip strength (kg)**		**Arm curl test in 30 seconds (#)**		**Chair stand test in 30 seconds (#)**		**Time 8**-**foot up and go test (secs)**		**Sit and reach (cm)**		**SF 36 physical health**	
	^*^Beta	**P**	^*^Beta	**P**	^*^Beta	**P**	^*^Beta	**P**	^*^Beta	**P**	^*^Beta	**P**	^*^Beta	**P**
Age	-0.032	.783	0.124	.290	0.274	.024	0.269	.025	-0.224	.046	-0.237	.060	0.192	.120
^a^Gender	0.070	.470	0.090	.363	0.112	.261	0.009	.930	0.016	.868	-0.042	.703	0.107	.302
^b^Marital status	0.098	.377	-0.263	.018	0.021	.852	0.003	.977	-0.017	.872	-0.038	.752	0.084	.302
**Education:**														
^c^High school	-0.125	.235	-0.063	.545	0.215	.043	0.112	.294	-0.299	.004	-0.075	.752	0.197	.069
^c^ > High school	0.158	.192	0.082	.498	0.127	.303	0.193	120	-0.328	.006	0.013	.924	0.142	.278
^**d**^**Income:**														
$15,000-$30,000	-0.031	.766	-0.63	.545	-0.112	.291	0.096	.361	0.139	.165	-0.066	.564	0.050	.642
>$30,000	0.071	.513	-0.052	.630	-0.024	.303	-0.060	.592	0.241	023	0.136	.266	-0.092	.426
**Ethnicity:**														
Chinese origin	-0.062	.651	0.134	.328	0.079	.572	0.327	.019	-0.248	.06	-0.04	.761	0.140	.313
^e^TC attended	-0.004	.973	-0.118	.328	-0.049	.648	0.038	.723	0.018	.858	-0.025	.827	-0.173	.111
^f^Previous TC experience	-0.153	.204	-0.118	.329	-0.068	.587	-0.047	.702	0.046	.688	-0.038	.769	-0.070	.569
^g^Physical activity	-0.223	.036	0.057	.577	-0.073	.479	0.056	.588	0.065	.504	-0.004	.974	0.058	.579
^h^Multi-morbidity	0.066	.506	0.009	.929	0.036	.716	-0.027	.784	0.120	.202	-0.054	.616	0.011	.913
R^2^	.166		.130		.107		.102		.177		.069		.086	

^a^Reference group male.

^b^Reference group single.

^c^Reference group < High School.

^d^Reference group < $15,000.

^e^Based on Overall Attendance.

^f^Tai Chi experience of one year or more.

^g^Based on Average Weekly Physical Activity.

^h^Reference group 1 or none chronic diseases.

*Standardized Beta.

Note: # designates repetitions completed.

## Discussion

This study examined the effect of a 16 week TC program on musculoskeletal health-related fitness changes and self-reported physical health in a community based program with low income mid to older adults from multiple ethnicities of origin. This study is the first TC study in Canada to look at a community-based program in this specific population. Results showed that a 16 week program aimed at 2 sessions per week participation has the potential for being beneficial on improving health-related musculoskeletal fitness and SF 36 measures, results could not be explained by traditional determinants of health. These benefits may be particularly valuable given that many participants attendance averaged less than 2 sessions a week, yet still showed improvements in health-related musculoskeletal measures and self-perceived physical health.

Research in community based settings often deal with complexities of health challenges (as well as environmental challenges) that can influence the research goals and program practices [21]. Factors such as geography, time of year, population density, population demographics plus cultural influences combine to create different and unique cohort influences. In this study multiple characteristics differed at baseline. Cohort 1 was based in a Toronto Community Housing building with an onsite auditorium while cohort 2 and 3 were both based in community centers that needed some participants to use some form of transportation to attend. Cohort 2 in the Chinese community had a wider age range of participants that used the community center and were motivated to partake in multiple social programs throughout the day. Toronto can have a variety of weather that can also influence attendance to any community program. Cohort 1 occurred summer into fall, whereas cohort 2 took place between spring into summer and cohort 3 occurred fall into winter. However despite these influences, the TC program showed consistent improvements across all cohorts in both health-related musculoskeletal fitness and SF 36 summary physical health measures.

The ability of aging adults to maintain quality of life through activities of daily living is important in a country that has an ever increasing maturing population [22]. To enhance health and empower self-management in this area accessible PA is an important factor for creating and sustaining well-being at all ages and especially so in aging adults [23]. The basic body functions, such as strength, endurance, balance and flexibility in upper and lower extremities, are all important to maintain physical independence in older age [24]. Overall grip strength has been found to be a reliable tool for the predictor of mortality, disability, health complications and length of hospital stay [25]. Multiple TC studies have found an increase in overall hand-grip strength [26] as did this present study. Similarly, evidence continues to build on the beneficial effects of musculoskeletal fitness in the prevention of chronic diseases and in combination with performance of activities of daily living [27]. Upper and lower body musculoskeletal fitness is important in executing many normal everyday activities such as household chores, carrying groceries, lifting objects and picking up grandchildren [24] as well as performance variables such as gait, stair climbing, rising from a chair, and balance [28]. Multiple TC studies have also shown upper and lower body strength improvements in older adults in line with our findings [29,30]. In Canada the fall-related injury rate is nine times greater among seniors than among those less than 65 years of age [31] thus the importance to prioritize programs that improve balance, mobility and strength. The timed 8 foot Up and Go test was specifically designed to test motor agility and dynamic balance for older adults [24]. In our study there was a significant improvement for the timed 8 foot Up and Go test similar to recent TC studies [32]. As older adults age their range of motion decreases and insufficient hamstring flexibility is associated with low back pain, increased susceptibility to injury and increased risk of falling [24]. Once again in our study there was a significant change in flexibility similar to other findings in the TC literature [33]. Finally when looking at the psychometric based physical scales from the SF 36 and the pre post differences in our study we found that physical function (PF), general health (GH) and the overall physical health summary measure (PCS) were significant. Our findings were similar with other Tai Chi studies that also showed significant changes in the physical scales of the SF 36 [34].

Even though this present study was a unique combination of low SES with multiple ethnicities there have been some TC studies that have used low SES populations. One study was done in a community center based in the United States with a lower income Chinese ethnicity of origin population [29]. This group of 39 mixed gender older adults were enrolled in a 12 week Yang style TC program and demonstrated significant results in muscular strength similar to our study [29]. Another group of low income Caucasian seniors living in the United States were enrolled in a 6 month TC study that focused on physical functioning as the primary outcome [35]. This study also showed significant improvements in all aspects of physical functioning [35].

Since this was a preliminary analysis targeting multiple ethnicities of origin and a low SES population, our goal was to explore potential relationships while attempting to control for known confounders. In this present study we found that no overall independent variables were strong predictors of health-related musculoskeletal fitness and SF 36 changes, however there were select individual variables that had predictive influences. It is also important to note that despite select significant variables, R^2^ values in the multivariate linear regression models were consistently small throughout all models. However since the duration of the program was only 4 months this was possibly too short a time to fully discover potential mediators. Future studies should be designed specifically to explore these mediators further. The few predictors that showed some potentially small influential effects such as initial physical activity levels, marital status, multi-morbidity and age are worth some consideration. As community PA programs continue to grow all populations, especially older adults, can increase their health benefits when participating in these programs even with small changes in BMI regardless of their initial PA levels [36]. Although research has explored marital status and longevity [37] the relationship between marital status and grip strength would be interesting to explore in futures studies. Grip strength has become a powerful tool for predicting frailty and increased risk for early morbidity and mortality and even despite increases in chronic conditions, as seen in our sample population, strength can be improved [25]. Finally as the population ages it is important to understand that this increase in years does not mean that strength, agility and power cannot be positively modified by physical activity programs like TC and that increased age also highlights the importance of physical activity programs as an anti-aging intervention [38]. In our analysis no predictors were associated with the sit and reach test and the summary measure of physical health in the SF 36. Despite the absence of strong predictors this could potentially point in the direction of the broad adaptability of TC on the musculoskeletal system. Since TC is a complex, multicomponent intervention that integrates effects on multiple systems [39] being unable to narrow outcomes to specific predictors could simply be reinforcing its strong multivariate influence.

Unlike a randomized controlled study, the current study design has known limitations related to internal validity of results. These limitations include uncontrolled program induced changes in daily physical activities, seasonal variations in health status and mood, lifestyle factors and self-reporting bias. However it should be noted the strength of this study are the real world outcomes demonstrated in a high at risk population that is under researched and over exposed to stressors from both aging and lower income.

## Conclusion

These findings suggest that a community based TC program with low income mid to older adult participants consisting of multiple ethnicities has the potential to be beneficial in improving health-related musculoskeletal fitness changes through strength, flexibility improvements and SF 36 physical scales. This program was effective with a wide range of participants with multiple chronic conditions ranging from metabolic to orthopedic that influenced a large range of functional abilities. It is important to note that a community program such as this can be offered at a minimal cost making it an accessible and sustainable approach to maintaining and/or enhancing health-related fitness in a wide variety of participants.

## Competing interests

There are on financial competing interests. No stocks or shares are held that are associated with this publication. No currently applying patents are involved. There are no non-financial competing interests.

## Authors’ contributions

JM: Enrolment of participants, data collection, data analysis, write-up of manuscript. MR: Statistician lead and critical revision of the manuscript for important intellectual Content. VJ: Research methods and critical revision of the manuscript for important intellectual Content. CA: Design of the project, research methods and critical revision of the manuscript for important intellectual Content. HT: Conception and design of the project, critical revision of the manuscript for important intellectual Content. All authors read and approved the final manuscript.

## Pre-publication history

The pre-publication history for this paper can be accessed here:

http://www.biomedcentral.com/1471-2318/13/114/prepub
